# Robotic-Assisted Total Knee Arthroplasty for Distal Femur Fracture with Lateral Knee Osteoarthritis

**DOI:** 10.1155/2021/5576955

**Published:** 2021-04-27

**Authors:** Takao Kaneko, Tadashi Igarashi, Shu Yoshizawa, Kazutaka Takada, Hiroyasu Ikegami, Yoshiro Musha

**Affiliations:** ^1^Ichinomiya Onsen Hospital, Adult Reconstruction Center, Japan; ^2^Department of Orthopedic Surgery, Toho University School of Medicine, Japan

## Abstract

*Introduction.* Open reduction and internal fixation (ORIF) of compound fractures around the knee in elderly patient raise concerns about long-term postoperative external fixation and complications. Total knee arthroplasty (TKA) has been proposed as an alternative solution. We report a case where robotic-assisted (RA) TKA was used to treat lateral knee osteoarthritis (OA) with distal femur fracture. *Case Presentation*. A 90-year-old female visited our hospital with complications of sustained knee pain after a fall at home. Fracture line from the trochlea to the intercondylar notch was diagnosed on plain radiographs, and prior to this injury, the patient was receiving conservative treatment for lateral OA. We selected a conventional TKA over ORIF because the latter is associated with residual pain and the need for long-term immobilization, which can lead to other complications. However, the fracture site was the entry point for intramedullary rod, and there was concern that the fracture site would be displaced by conventional TKA. Therefore, the unique aspect of the case is that the technique utilized involved robotic milling using the Navio system while temporarily stabilizing the fracture using two tracker pins. RA TKA could determine osteotomy and implant placement by predicting the postoperative patient's soft tissue balance for no medial loosening and lateral contracture. The arthritic cartilage and bone were then methodically removed using the handheld sculptor. After immobilizing the fracture site with a bone grasper before removing the pin tracker, reaming of the femur and insertion of a stem prosthesis with semiconstrained were performed. Primary RA TKA is a viable option for intra-articular fractures in elderly patients with advanced knee osteoarthritis.

## 1. Introduction

Open reduction and internal fixation of compound fractures around the knee in elderly patients with osteoporosis have resulted in poor outcomes due to prolonged postoperative immobilization. Arthroplasty is commonly used to treat acute fractures of the proximal humerus, elbow, or proximal femur [1, 2] but is less commonly used for complex knee fractures [3, 4]. The main goal of total knee arthroplasty (TKA) in distal femur fractures is to save the patient's quality life by avoiding complications and other problems, thanks to the immediate resumption of weight bearing. Current developments in robotic-assisted surgical techniques have given surgeons intraoperative options to improve accuracy [5, 6]. There have been no reports of primary robotic-assisted TKA for fractures around the knee in elderly patients. We present a case of robotic-assisted (RA) TKA for lateral knee osteoarthritis with distal femur fracture.

## 2. Case Presentation

**Figure 1:**
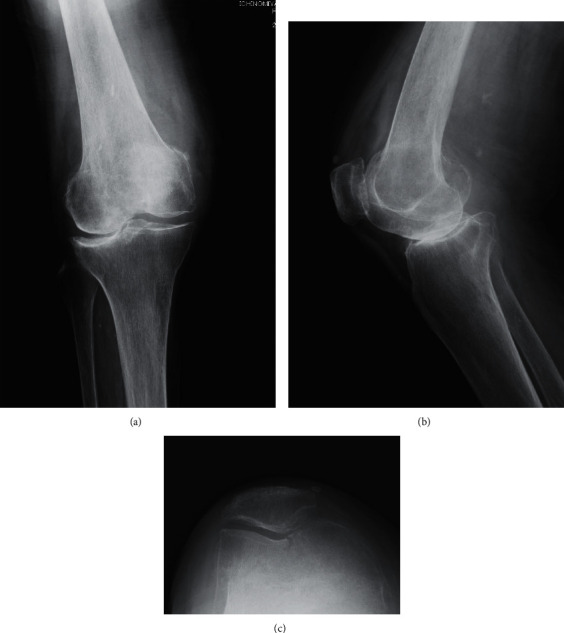
A 90-year-old female with a fracture of the distal femur with lateral knee osteoarthritis. (a) AP view, (b) lateral view, and (c) axial view.

**Figure 2:**
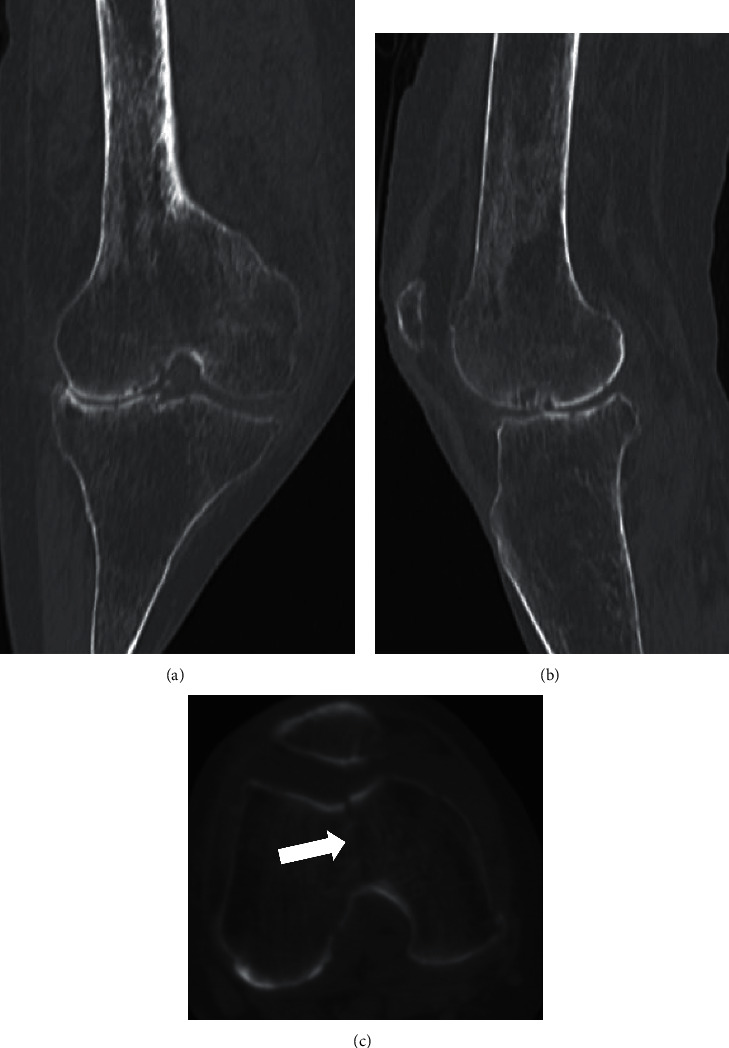
Two dimensional computed tomography (2DCT) scan demonstrating a fracture line from the trochlea to the femoral groove (AO Foundation/Orthopedic Trauma Association classification: type B2) (white and black arrow). (a) Coronal view, (b) sagittal view, and (c) axial view.

A 90-year-old woman with a body mass index (BMI) of 22.1 kg/m^2^ visited our hospital with complaints of right knee pain after a fall at home that made it difficult for her to walk. In the emergency department, the patient exhibited swelling of the right knee and tenderness over the distal femur. Plain radiographs of the right knee showed a fracture of distal femur with lateral knee osteoarthritis (Figure 1). Two-dimensional and three-dimensional computed tomography (CT) revealed a fracture line from the trochlea to the intercondylar notch (AO Foundation/Orthopedic Trauma Association classification type B2) (Figure 2). Prior to this injury, the patient had been treated at another hospital for lateral knee osteoarthritis (OA) (Krackow classification type I [7] (Figure 1) with conservative treatments such as intra-articular injections, but there were no signs of improvement. We selected a primary RA TKA over open reduction and internal fixation because the latter is associated with residual pain and the need for long-term immobilization, which can lead to other complications.

### 2.1. Surgical Procedure

TKA was performed with the medial subvastus approach. A robotic-assisted system was used in the image-free handheld RA surgery with the Blue Belt Navio surgical system (Navio; Smith & Nephew, Plymouth, MN, USA) [5, 6].

**Figure 3:**
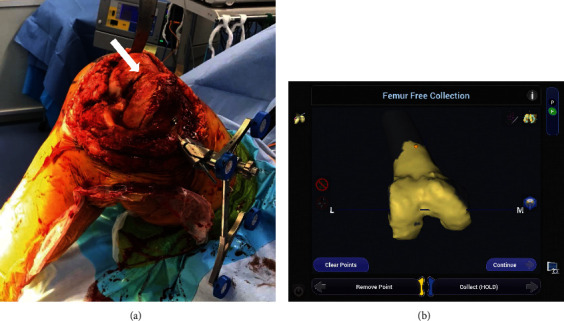
The fracture line (white arrow) is fixed using two bury pin threads (4.0 mm) and anatomy of the femoral condyle were mapped by “painting” the surfaces with an optical probe. The surface was coated with an optical probe, but it was reduced and there was no separation of the fracture. (a) Intraoperative photograph and (b) image free registration.

**Figure 4:**
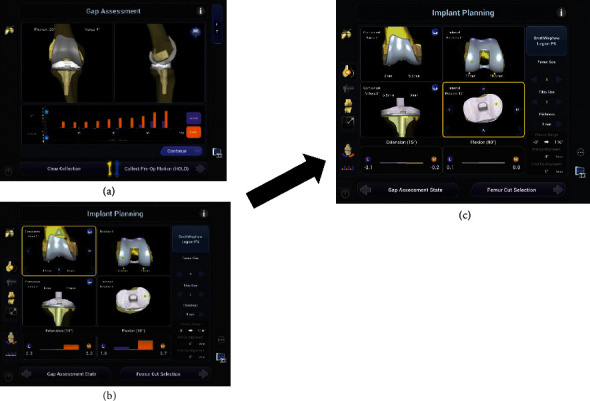
Patient-specific planning. (a) A varus/valgus stress test was performed with a manual max through a full range of motion. (b) The medial gap was enlarged with flexion, and mild lateral contracture was observed. Due to reduction as much medial looseness from extension to flexion and lateral contracture from extension to midflexion, femoral and tibial components were performed in valgus alignment and we fine-tuned the amount of osteotomy. (c) Medial and lateral gaps became constant.

A fracture line was observed from the trochlea to the intercondylar notch. The fracture site coincided with the entry point for the intramedullary rod at the time of the conventional TKA. Temporary fixation with two bury pin threads (4.0 mm) was initially used to fix the fracture line (Figure 3), and tracker fixation of the tibia was performed from the anteromedial tibia to 6 cm from the incision. The osteophytes on the femur and tibia were resected. Landmark registration and anatomy of the femoral condyle and tibial plateau were mapped by “painting” the surfaces with an optical probe without separation of the fracture site (Figure 3). A varus/valgus stress test was performed with a manual max through a full range of motion. The medial gap opened through a full range of motion, and mild lateral contractures were noted in extension to midflexion. Prosthesis size (femoral component: 4, tibial component: 3), alignment (femoral and tibial component valgus aligned), positioning, and volume of bone removal were determined intraoperatively by the surgeon (Figure 4). As a result, soft tissue balance (extension—medial: -0.2 mm, lateral: -0.1 mm; flexion—medial: 0 mm, lateral: 0.1 mm) was identified (Figure 4). No loosening of the medial soft tissue was observed at extension and 90 degrees of knee flexion (Figure 4). In other words, the planning of prosthesis positioning and bone resection occurred intraoperatively with consideration of soft tissue balance.

**Figure 5:**
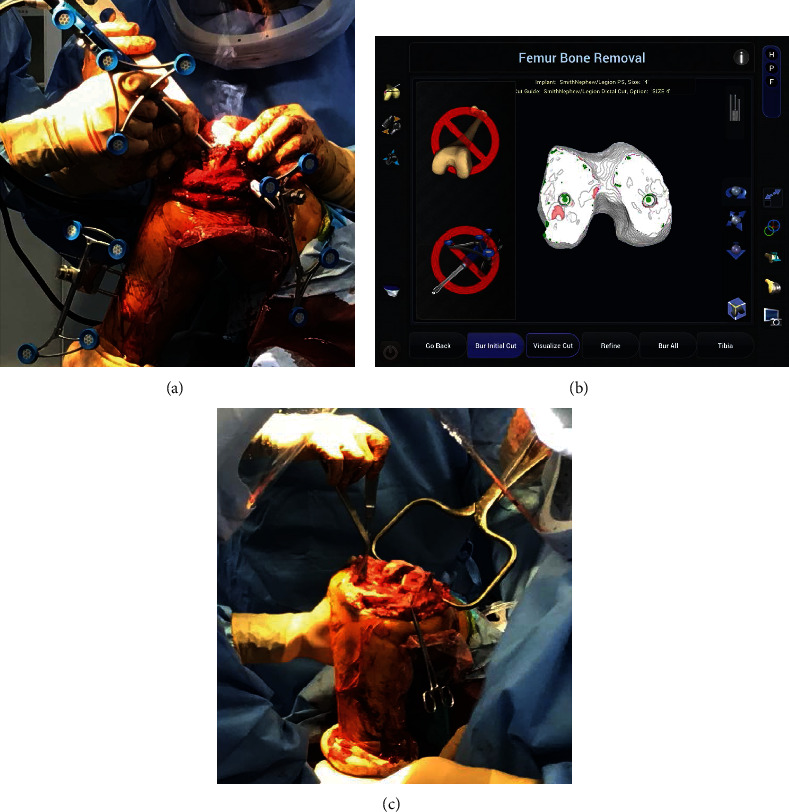
(a) Intraoperative photograph. (b) Navio screen allowing the surgeon to continually assess the patient anatomy against the plan. The articular cartilage and bone being removed using a hand-held sculptor. Green: approximately 1 mm or more and less than from target surface; white: near the target surface (less than 1 mm); red: below the target surface. (c) After immobilizing the fracture site with a bone grasper before removing the pin tracker, reaming of the femur and insertion of a stem prosthesis were performed.

**Figure 6:**
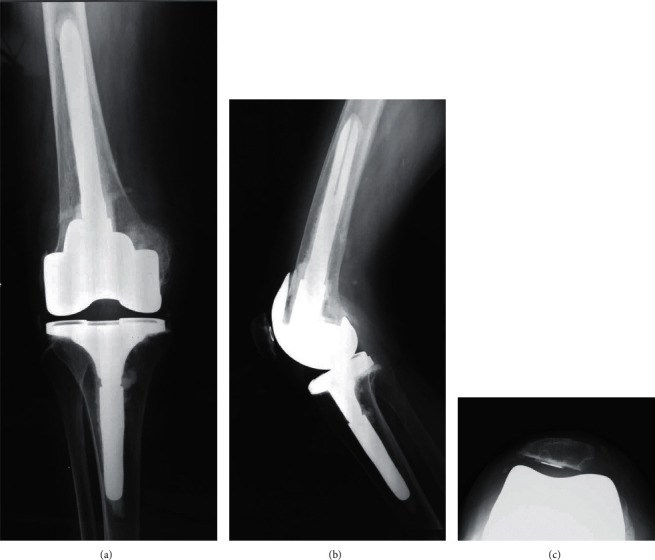
Postoperative radiographs showing total knee arthroplasty using the semiconstrained lesion prosthesis (Smith & Nephew). (a) AP view, (b) lateral view, and (c) axial view.

The arthritic cartilage and bone were then methodically removed using the handheld sculptor while holding the medial and lateral condyles of the femur, to prevent the fracture site from separating (Figure 5). During the RA TKA, the position of the patient's lower extremity and the progress of bone resection was continuously tracked using a navigation system camera. The medial gap looseness was improved from the intraoperative balancing procedure, and resurfacing TKA could have been chosen. However, the semiconstrained lesion prosthesis (Smith & Nephew, Memphis, TN) (femoral component—4 (stem extension; 12 mm × 160 mm); tibial component—3 (stem extension; 12 mm × 100 mm)) was chosen to ensure the stability of the fracture (Figure 6). After immobilizing the fracture site with a bone grasper before removing the pin tracker, reaming of the femur and insertion of a stem prosthesis were performed (Figure 5). The operative time and blood loss were 124 minutes and 105 ml, respectively.

On the two days after TKA surgery, the drainage tube was removed and physical therapy was initiated. A physical therapist started isometric strength exercises for the quadriceps and ROM exercises for the knee joint. Full weight-bearing was not restricted, and the patient was allowed to walk with or without assistive devices.

Prosthetic alignment, Knee Society Scores, and range of motion were assessed at 12 months after surgery (Table 1).

## 3. Discussion

We performed a primary RA TKA for a fracture of the medial condyle of the distal femur with lateral knee osteoarthritis. There are several advantages to this technique. First, two bury pin threads allowed us to fix the fracture site in place and easily burring the distal femur. Second, by using the Navio system, varus or valgus stress is applied to tension the soft tissues on the sides of the knee through a full range of flexion to plan the desired soft tissue laxity. This helps the surgeon plan for implant positioning and volume bone resections, taking into account “virtual” soft tissue laxity prior to making any cuts [8–11]. We were likewise able to determine the positioning of the prosthesis and the plan for osteotomy intraoperatively to ensure that there was no medial laxity, while taking soft tissue balance into account. Elderly patients with osteoporosis and osteoarthritis prior to the fracture are the most frequently encountered patient group [3, 4]. These patients present emergently with a complex comminuted articular fracture of the distal femur or proximal tibia. X-ray findings include signs of osteoarthritis, and the history often reveals that the patient was already suffering severe pain before the fracture. Arthroplasty may in some cases have already been scheduled by another surgeon before the fracture occurred. In the situation, arthroplasty is a logical solution for treating the fracture and osteoarthritis [3, 4, 12, 13].

Krachow et al. [7] classified valgus deformity of the knee into three types. Type I was defined as valgus deformity secondary to bone loss in the lateral compartment and soft-tissue contracture with intact medial soft tissue. Type II was defined as obvious attenuation of the medial capsular ligament complex. Type III was defined as severe valgus deformity with valgus malpositioning of the proximal tibial joint line after proximal tibial osteotomy with overcorrection. The present case was not passively correctable with the varus test. There has been a report of revision surgery due to medial soft tissue laxity [14]. In Krachow classification type II, medial looseness may persist even with minimal osteotomy. If the medial soft tissue is loose and releases laterally, the joint space may widen more than expected, so the tibia should be osteotomized 6 to 9 mm from the medial condyle [15, 16].

A case report has advantages. First, regarding the rationale for the use of robots in this case, the fracture was nondisplaced, and while a traditional cutting block could have been used, with the help of a large reduction clamp and/or wire, there were advantages to using robotic milling performed with minimal applied force to the distal femoral cartilage surface without inserting an intramedullary rod by fixing the fracture with a tracker pin. Second, RA TKA is a viable option for intra-articular fractures in elderly patients with advanced knee osteoarthritis. Third, physical therapy, which was initiated immediately postoperatively, included full weight-bearing without restriction. Fourth, RA TKA achieves good clinical outcomes at 1 year postoperatively despite advanced age.

A case study has disadvantages. First, we pulled out the 4.0 mm bury pin threads to insert the prosthesis with the stem. Consequently, we have not been able to verify intraoperative kinematics. Second, Renawat et al. [15] reported that the angle between the mechanical alignment and the anatomical alignment should normally be set at 6 degrees but always at 3 degrees to prevent undercorrection. However, the valgus angle was 5 degrees to prevent loose medial soft tissue balance in this patient. Third, femoral rotation alignment was 2 degrees of external rotation and tibial internal rotation angle was 11 degrees of internal rotation, resulting in rotational mismatch. For the valgus knees, it has been reported that the posterior condylar axis of the femur is approximately 5 degrees more internally rotated than the varus knee, due to hypoplasia of the lateral epicondyle of the femur [17]. We were concerned that more than 3 degrees of external rotation of the posterior condylar axis of the femur would result in looser medial soft tissue balance during flexion. The internal rotation angle of the tibia was determined from the perspective of prosthesis coverage based on the bone morphology of the tibia. We need to pay attention to the mechanical stress that will be added to the post and cam of the prosthesis. Fourth, in this case, excessive tests such as DXA test for osteoporosis were not performed due to posttrauma. Fifth, RA TKA was performed, which costs a lot of time and money for the surgery, adding additional risk.

The unique aspect of the case is that the technique utilized involved robotic milling using the Navio system while temporarily stabilizing the fracture using the tracking pin.

In conclusion, primary RA TKA is a viable option for intra-articular fractures in elderly patients with advanced knee osteoarthritis.

## Figures and Tables

**Table 1:** Pre- and postoperative clinical data (90-year-old female, fracture).

Preoperative		Postoperative	

*1989 Knee Society Score*		*Prosthetic alignment*	
Pain (50)	0	*α* angle (°)	96.4
Knee (97)	0	*β* angle (°)	89.7
Function (100)	-20	*γ* angle (°)	2.1
		*δ* angle (°)	90
		Femoral tibial angle (°)	175
		*Knee Society Score*	
		Symptom (25)	18
		Patient satisfaction (40)	30
		Patient expectation (15)	13
		Activity (100)	71
		*Range of motion*	
		Extension angle (°)	5
		Flexion angle (°)	120

## Data Availability

There are no available data.
